# ABO Blood Groups, Lipids, and Coronary CT Imaging in A Japanese Single-Center Cohort

**DOI:** 10.3390/medsci14010121

**Published:** 2026-03-04

**Authors:** Hiroyuki Tokue, Azusa Tokue, Yoshito Tsushima

**Affiliations:** Department of Diagnostic and Interventional Radiology, Gunma University Hospital, 3-39-22 Showa-Machi, Maebashi 371-8511, Gunma, Japan

**Keywords:** ABO blood group, HDL-cholesterol, coronary artery calcium, coronary CT angiography, hypertension

## Abstract

**Background and Objectives:** Non-O ABO blood groups have been linked to higher coronary risk, plausibly via hemostatic and lipid pathways. However, evidence in Japanese populations and imaging-defined disease is limited. We examined whether ABO status relates to serum lipids and coronary CT imaging findings in Japanese adults. **Materials and Methods:** We reviewed adults who underwent coronary CT angiography (CCTA) at our institution. After prespecified exclusions, 865 patients comprised the imaging cohort. For lipid analyses, we excluded patients receiving lipid-lowering therapy at the time of blood sampling, leaving 636 patients (lipid subset). ABO blood group was obtained from the medical record as recorded at registration (patient-reported) and was not re-confirmed by laboratory testing for this study. Outcomes were any coronary artery calcium (Agatston score > 0) and ≥50% luminal stenosis on CCTA. **Results:** In the lipid subset (*n* = 636), coronary calcium was present in 44–54% of patients across the four ABO groups and did not differ across groups (*p* = 0.33). Among assessable scans in the imaging cohort, ≥50% stenosis did not differ across the four ABO groups. In multivariable models (*n* = 636), older age, male sex, hypertension, and diabetes were independently associated with both outcomes (CAC presence and ≥50% stenosis) (all *p* < 0.05). For ≥50% stenosis, higher High-Density Lipoprotein-cholesterol (HDL-C) was additionally associated with lower odds (*p* < 0.05). ABO status (O vs. non-O) was not independently associated with either outcome. **Conclusions:** In Japanese adults undergoing CCTA, type O blood was tied to lower HDL-C and higher diastolic pressure—features that track with cardiometabolic risk—yet ABO type did not independently relate to coronary calcium or CT-defined stenosis once standard risk factors were considered. These data suggest that, in this setting, ABO adds little beyond conventional risk profiling.

## 1. Introduction

The ABO blood group system has long featured in cardiovascular epidemiology, with meta-analyses suggesting that non-O groups carry higher coronary risk than group O [1]. Proposed mechanisms include higher circulating von Willebrand factor and factor VIII as well as unfavorable lipid traits among non-O individuals [2]. Yet the literature is dominated by Western and Chinese cohorts and typically emphasizes clinical events rather than imaging-defined disease. Whether these associations generalize to Japanese populations—and whether ABO status maps onto contemporary coronary CT imaging findings—remains an open question.

Beyond coronary events, ABO status has been linked to a broad spectrum of cardiometabolic and thrombotic phenotypes across populations [3]. Large prospective cohorts have reported higher risk of coronary heart disease among non-O blood groups, with consistent directions of association in both women and men [2]. A recent narrative review further summarized evidence linking non-O blood groups to increased risk and severity of coronary artery disease, potentially through hemostatic, lipid, inflammatory, and endothelial pathways [4]. Mechanistically, ABO is a major determinant of von Willebrand factor (vWF) and factor VIII levels, with blood group O typically showing ~20–30% lower levels, which may translate into lower thrombotic risk [5]. In parallel, genetic and epidemiologic studies suggest that part of the ABO–coronary association may be mediated through lipid traits and other cardiometabolic pathways [6].

However, most prior evidence has focused on clinical endpoints, while fewer studies have examined imaging-defined coronary atherosclerosis. Coronary artery calcium (CAC) and plaque/stenosis phenotypes may capture earlier or subclinical disease and provide mechanistic insight beyond event-based studies. Prior investigations in non-Japanese cohorts have reported mixed results regarding ABO and imaging markers, including CAC and plaque characteristics, highlighting the need for population-specific evaluation [7]. In large population-based data, associations between ABO blood groups and vascular outcomes have not been uniform; for example, a nationwide Swedish cohort found no significant association between ABO blood group and aortic aneurysm or dissection [8].

Notably, older Japanese data suggested ABO-related differences in total cholesterol distribution within Japan, supporting the possibility that ABO–lipid relationships may vary by ancestry and environment [9]. Japan differs from Western settings in ABO distribution, dietary patterns, and cardiometabolic background, all of which may shape lipid traits and the hemostatic milieu. Despite the routine use of coronary CT angiography (CCTA) in Japan, evidence linking ABO type to coronary artery calcium (Agatston score) or CT-defined luminal stenosis in Japanese adults is sparse. CCTA provides a quantitative window into plaque burden and stenosis with high diagnostic performance for ≥50% lesions and plays a central role in guideline-endorsed coronary artery disease (CAD) assessment [10,11]. It therefore offers a suitable framework to test whether ABO status confers information beyond established risk factors. Recent large-scale studies have reported associations between ABO alleles and cardiometabolic outcomes, including myocardial infarction [12].

In this single-center cohort of Japanese adults referred for CCTA (final analytic *n* = 865), we compared conventional risk factors and serum lipids across ABO groups and evaluated whether ABO status independently associated with (i) any coronary calcification and (ii) significant stenosis on CCTA after multivariable adjustment.

## 2. Materials and Methods

### 2.1. Study Design and Ethics

We conducted a single-center retrospective cohort study using clinical and imaging data acquired during routine care between 2015 and 2024. The study protocol was approved by the Institutional Review Board of Gunma University Hospital (approval no. HS-2025-231; approval date: 20 November 2025) and complied with the Declaration of Helsinki and the STROBE statement [13]. Written informed consent was waived, and an opt-out procedure was implemented as approved by the IRB.

### 2.2. Patient Selection

Between 2015 and 2024, 1300 patients underwent CCTA at our hospital for chest symptoms, electrocardiographic abnormalities, or a positive treadmill test. We excluded patients with (1) a prior history of coronary heart disease; (2) missing ABO blood group or missing laboratory measurements required for the primary imaging analyses within 3 months (e.g., glucose or creatinine); (3) nondiagnostic image quality due to motion or metal artifacts; and (4) non-Japanese nationality. For lipid comparisons across ABO groups, we additionally excluded patients receiving lipid-lowering therapy at the time of blood sampling. Participants were classified into four ABO groups (A, B, AB, and O). The prespecified primary comparison was group O versus non-O (A/B/AB combined); four-group results are presented to describe exposure categories.

### 2.3. Definitions of Risk Factors

Family history of coronary heart disease was defined as a first- or second-degree relative with documented disease. Hypertension was defined as systolic blood pressure ≥ 140 mmHg and/or diastolic blood pressure ≥ 90 mmHg, or current antihypertensive treatment. Diabetes mellitus was defined as fasting plasma glucose ≥ 126 mg/dL, postprandial glucose ≥ 200 mg/dL, HbA1c ≥ 6.5% (48 mmol/mol), or use of glucose-lowering medication. Dyslipidemia was defined as HDL-cholesterol (HDL-C) < 40 mg/dL, LDL-cholesterol (LDL-C) > 140 mg/dL, triglycerides > 150 mg/dL, or use of lipid-lowering medication. Smoking status was defined as any smoking within the previous year. Chronic kidney disease was defined as an estimated glomerular filtration rate (eGFR) < 60 mL/min/1.73 m^2^ (KDIGO criterion) [14]. Serum creatinine was also recorded.

### 2.4. CT Hardware and Acquisition Protocol

All scans were performed on a 320-row multidetector CT system (Aquilion ONE ViSION Edition; Canon/Toshiba Medical Systems, Otawara, Japan; 0.275 s rotation). Continuous ECG monitoring (IVY3000, Chronos Medical Devices, Chiba, Japan) and a dual-syringe power injector (Stellant Dual Flow, Nihon Medrad, Osaka, Japan) were used.

Heart-rate control: For scheduled examinations, patients with resting heart rate (HR) ≥ 61 bpm received oral bisoprolol 2.5 mg 2–4 h before scanning unless contraindicated. If HR control was suboptimal, intravenous propranolol (2–10 mg) was administered immediately prior to acquisition. For same-day add-on studies, intravenous propranolol (2–10 mg) or landiolol (0.125 mg/kg) was given at the operator’s discretion.

Calcium scoring (non-contrast): A non-contrast scan from the sinotubular junction to the left ventricular apex was obtained in all patients for coronary artery calcium scoring (CACS). Reconstructions used 3.0 mm slices. Agatston scores were computed on a dedicated workstation (Ziostation 2; Ziosoft, Tokyo, Japan) following the original method [15].

Contrast-enhanced CCTA: Prospectively ECG-triggered one-beat volume scans were the default. Data were reconstructed primarily in mid-diastole. If the diastolic rest period was insufficient, end-systolic reconstruction was used. Nominal collimation was 0.5 mm; images were reconstructed at 0.5 mm thickness with 0.25 mm increment using a medium-sharp cardiac kernel. Tube voltage was 120 kV by default; tube current was modulated with automatic exposure control (Volume EC) targeting an image noise SD of 20 HU. Effective dose was estimated from the dose–length product using a chest conversion factor (k = 0.014 mSv·mGy^−1^·cm^−1^).

Contrast injection and bolus timing: A nonionic, low-osmolar iodinated contrast agent (iopamidol 300–370 mg I/mL; market-approved brand) was administered via an antecubital vein using a weight-based rate of body weight × 0.06 mL/s for 11 s, followed by saline at the same rate for 8 s. Bolus tracking was performed with the region of interest in the left ventricular cavity; acquisition commenced 7 s after visual confirmation of contrast arrival.

### 2.5. Image Analysis and Study Outcomes

CACS was analyzed as a binary endpoint (Agatston > 0 vs. 0) for primary analyses, with exploratory categories (0, 1–99, 100–399, ≥400). Coronary stenosis was assessed on CCTA using a 17-segment AHA model by experienced readers blinded to ABO type. Significant stenosis was defined a priori as ≥50% diameter narrowing in any epicardial segment. In sensitivity analyses, we applied a ≥70% threshold. Discrepancies were adjudicated by consensus. Image quality and motion/metal artifacts were graded; studies rated nondiagnostic were excluded per Section 2.2.

### 2.6. Laboratory Measurements

ABO blood group was obtained from the medical record as recorded at registration (patient-reported). Lipid profiles (LDL-C, HDL-C, triglycerides) and other laboratory values (glucose and creatinine) were measured in serum/plasma samples in the central hospital laboratory as part of routine clinical care, using standardized enzymatic assays on an automated analyzer. Laboratory values obtained within 3 months of the CCTA examination were extracted from the medical record; when multiple results were available, the value closest to the CCTA date was used.

### 2.7. Statistical Analysis

Continuous variables are presented as mean ± standard deviation (SD) when approximately normally distributed; skewed variables (e.g., Agatston score) are presented as median. For comparisons across the four ABO groups (A, B, AB, and O), we used one-way ANOVA for approximately normally distributed continuous variables and the Kruskal–Wallis test for non-normally distributed variables. We report global *p*-values for overall group differences. When the overall four-group comparison was significant (*p* < 0.05), post hoc pairwise comparisons were performed using Tukey’s honestly significant difference (HSD) test for continuous variables.

Categorical variables were compared using the chi-square test or Fisher’s exact test, as appropriate. When the overall four-group comparison for a categorical variable was significant, pairwise comparisons were performed using the chi-square test (or Fisher’s exact test, as appropriate). Because only a limited number of categorical variables showed significant overall differences, these pairwise comparisons were reported for transparency and interpreted descriptively.

To assess the association of ABO group with coronary calcification and luminal stenosis, multivariable logistic regression was performed. Multivariable logistic regression analyses were restricted to the lipid subset (*n* = 636), excluding patients receiving lipid-lowering therapy at the time of sampling, to allow adjustment for measured lipid levels with reduced treatment-related confounding. Two-sided *p* < 0.05 was considered statistically significant. A commercially available software package (SPSS Statistics, v22, IBM, Armonk, NY, USA) was used for all analyses.

## 3. Results

### 3.1. Study Flow and Final Cohort

Between 2015 and 2024, 1300 adults underwent CCTA. After prespecified exclusions—prior coronary heart disease (*n* = 298), missing ABO type or required laboratory measurements within 3 months (e.g., glucose or creatinine) (*n* = 121), nondiagnostic image quality due to motion/metal artifacts (*n* = 5), and non-Japanese nationality (*n* = 11)—the final analytic cohort comprised 865 patients. For lipid comparisons, analyses were performed in patients with available lipid measurements within 3 months of CCTA and not receiving lipid-lowering therapy at the time of sampling (lipid subset, *n* = 636). The prespecified primary analyses compared blood group O versus non-O. In accordance with STROBE recommendations for reporting descriptive data by exposure categories, baseline characteristics and lipid/CAC measures are also presented across the four ABO groups (A, B, AB, O) for transparency (Table 1 and Table 2).

### 3.2. Patient Characteristics: Comparison Across Four ABO Groups

Baseline characteristics across the four ABO groups are summarized in Table 1, and lipid/CAC measures are shown in Table 2. In baseline characteristics (Table 1), DBP differed across ABO groups, with group O higher than group AB in exploratory pairwise comparisons (*p* = 0.034). The prevalence of hypertension also differed, with group O higher than group AB in exploratory pairwise comparisons (*p* = 0.032). Across the four ABO groups, HDL-C and the LDL/HDL ratio differed significantly (Table 2). Post hoc pairwise (Tukey-adjusted *p* values) comparisons showed lower HDL-C in group O than in groups A (*p* = 0.023), B (*p* = 0.032), and AB (*p* = 0.042). The LDL/HDL ratio was higher in group O than in groups A (*p* = 0.031) and AB (*p* = 0.012). No other pairwise comparisons reached statistical significance.

### 3.3. Primary Analysis: O vs. Non-O Comparisons

When each blood type was compared against the remaining types combined, A, B, and AB showed no consistent differences in blood pressure or lipids (all *p* > 0.10). In contrast, type O vs. non-O was associated with higher diastolic blood pressure, lower HDL-cholesterol, a higher LDL/HDL ratio, and a greater prevalence of hypertension (DBP and HDL-C, both *p* = 0.03–0.01; LDL/HDL *p* = 0.02; hypertension *p* = 0.04). Other risk factors were broadly similar between O and non-O (Table 3).

### 3.4. Coronary Artery Calcium

Across ABO groups, we found no material differences in the presence or burden of CAC (all *p* > 0.10; Table 2). Median Agatston scores were numerically higher in groups O and A than in groups B and AB, but differences were not statistically significant (Kruskal–Wallis *p* = 0.14), consistent with the null findings for any CAC (Agatston > 0).

In the O vs. non-O comparison, the median Agatston score was numerically higher in group O than in non-O (14.3 vs. 5.1), but this difference did not reach statistical significance (*p* = 0.20), likely reflecting the highly skewed distribution of Agatston scores with many zero values and a long right tail.

### 3.5. CT-Defined Significant Stenosis

Among assessable scans, ≥50% stenosis did not differ across ABO groups (global *p* > 0.10).

### 3.6. Multivariable Analyses: Impact of Blood Type on Calcification and Stenosis

In logistic models adjusted a priori for age, sex, BMI, smoking, hypertension, diabetes, LDL-C, HDL-C, triglycerides, and eGFR, age, male sex, hypertension, and diabetes were associated with any CAC and with ≥50% stenosis (all *p* < 0.05). ABO status (O vs. non-O) was not an independent predictor of either outcome; adjusted odds ratios were close to 1.0 with 95% CIs crossing 1.0 (Table 4 and Table 5). Sensitivity analyses (stenosis threshold ≥ 70%, sex/age stratification) gave consistent null associations for ABO status.

## 4. Discussion

In this Japanese single-center cohort of adults undergoing CCTA, blood group O was associated with lower HDL-cholesterol, a higher LDL/HDL ratio, and higher diastolic blood pressure compared with non-O groups. However, ABO status was not associated with coronary artery calcium (Agatston score > 0) or ≥50% stenosis on CCTA, and ABO (O vs. non-O) remained non-significant after multivariable adjustment for traditional risk factors. These findings suggest that, in this referral population, ABO status adds limited incremental value beyond conventional cardiometabolic risk factors for CT-defined coronary disease. Although the median Agatston score was numerically higher in group O, the between-group comparison was not statistically significant. Moreover, ABO status was not associated with CAC presence or CT-defined stenosis in adjusted models, suggesting that any ABO-related difference in calcification burden is small relative to established risk factors in this referral cohort.

Accumulating evidence suggests that ABO blood type is related to both the frequency and severity of diverse diseases such as cancer, inflammation, and metabolic disorders [16,17,18,19]. Substantial literature also implicates ABO status in cardiovascular disease, especially venous thromboembolism and ischemic heart disease, with non-O groups consistently exhibiting greater risk than group O [2,20,21]. Meta-analyses confirm non-O blood type as an independent predictor of CAD [22,23].

The leading explanation centers on ABO-encoded glycosyltransferases present in non-O groups [5]. ABO antigens influence glycan expression and have been discussed as potential biological underpinnings linking ABO status to vascular phenotypes [24]. These enzymes modulate von Willebrand factor and coagulation factor VIII; in non-O individuals, the vWF–FVIII complex has a longer half-life, raising circulating levels by about 25% [25]. This hemostatic shift is thought to promote venous thrombosis and, downstream, arterial atherosclerosis. A second, plausible pathway involves lipids: non-O status has been linked to higher total and LDL cholesterol [6]. ABO status may also interact with lipoprotein-related risk such as Lp(a) in coronary populations [26]. And a recent genome-wide association study estimated that ~10% of the ABO effect on CAD risk may be mediated by plasma cholesterol [1,27].

In the present study, we examined whether Japanese adults exhibit lipid profiles and CCTA-defined coronary phenotypes similar to those reported in previous studies. Contrary to expectations, our findings differed from those reported in cohorts from North America, Europe, West Asia, and China. In our cohort, individuals with blood group O exhibited lower HDL-cholesterol levels rather than higher LDL-cholesterol levels. They also had higher diastolic blood pressure, a feature that may reflect an unfavorable cardiometabolic profile. Although multivariable logistic regression did not demonstrate an independent association between ABO blood type and coronary calcification or stenosis, our findings suggest that blood type-related cardiometabolic characteristics in Japanese individuals may differ from those reported in other populations.

The reasons for these differences in Japan are not yet clear. A genetic contribution is plausible, since population genetics can influence cholesterol patterns. Interestingly, similar results have been reported in eastern India: Biswas et al. observed more group O among CAD cases and reduced HDL in group O [28]. In addition to ancestry, common lifestyle or dietary habits could affect lipid metabolism and thus CAD risk. Understanding these background factors may suggest new preventive or therapeutic approaches. Additional studies are required to resolve the discrepancy.

This study extends prior ABO–coronary research by evaluating a Japanese clinical CCTA cohort with standardized CT acquisition and blinded image interpretation, linking ABO status not only to serum lipid traits but also to imaging-defined coronary phenotypes (CAC and ≥50% stenosis). By prespecifying the primary comparison (O vs. non-O) while also reporting four ABO exposure categories, the analysis provides clinically interpretable contrasts and transparent exposure reporting. The results highlight potential population-specific ABO–lipid patterns and suggest limited incremental value of ABO status for CT-defined coronary disease beyond conventional risk factors in this setting.

There are some limitations in this study. This is a single-center, retrospective analysis with inherent selection and referral bias. ABO was self-reported, risking nondifferential misclassification. Lipids were measured within 3 months, but HDL functionality and apoB were not assessed; medication changes between blood draw and CCTA could confound interpretation despite planned exclusions for lipid-lowering therapy in the lipid analyses. Finally, generalizability to the broader Japanese population is uncertain.

Strengths of this study include (i) a relatively large single-center CCTA cohort with uniform CT hardware and protocol; (ii) blinded assessment of stenosis using a standardized segment model; (iii) evaluation of two complementary CT phenotypes (CAC and luminal stenosis) alongside lipid traits; (iv) a prespecified primary contrast (O vs. non-O) with multivariable adjustment for established risk factors; and (v) sensitivity analyses supporting the robustness of the null ABO–imaging associations.

## 5. Conclusions

In this Japanese referral cohort undergoing clinically indicated CCTA, blood group O was associated with lower HDL-cholesterol, a higher LDL/HDL ratio, and higher diastolic blood pressure and hypertension prevalence compared with non-O groups. However, ABO status was not associated with CAC presence or ≥50% CT-defined stenosis, and the O vs. non-O contrast remained non-significant after adjustment for conventional risk factors. These findings suggest that ABO status may correlate with certain cardiometabolic risk profiles but may add limited incremental value for CT-based coronary risk stratification in this setting. Prospective, multicenter studies with laboratory-confirmed ABO typing and expanded biomarker profiling are warranted to clarify population-specific mechanisms and clinical utility.

## Figures and Tables

**Table 1:** Baseline characteristics across ABO groups (*n* = 865).

Characteristic (n)	A (320)	B (182)	AB (95)	O (268)	*p*-Value
Age, years	67 ± 11	66 ± 12	67 ± 12	68 ± 11	0.54
Male, %	54	56	50	55	0.53
Height, cm	161 ± 9	160 ± 10	160 ± 10	160 ± 9	0.37
Weight, kg	62 ± 12	61 ± 13	61 ± 13	62 ± 13	0.31
BMI, kg/m^2^	24.1 ± 3.8	24.0 ± 4.0	23.2 ± 4.1	24.2 ± 3.9	0.39
SBP, mmHg	147 ± 23	146 ± 22	145 ± 21	147 ± 22	0.43
DBP, mmHg	86 ± 13	86 ± 13	85 ± 14	88 ± 14	0.030
Family history, %	27	26	23	28	0.25 ^†^
Hypertension, %	59	56	54	65	0.029 ^†^
Dyslipidemia, %	63	61	57	65	0.12 ^†^
Diabetes mellitus, %	22	21	19	21	0.58 ^†^
Smoking, %	16	15	14	13	0.26 ^†^
CKD, %	3	2	1	3	0.34 ^†^

**Abbreviations:** BMI, body mass index; SBP, systolic blood pressure; DBP, diastolic blood pressure; CKD, chronic kidney disease; ABO, ABO blood group. **Notes:** Data are mean ± SD or % unless otherwise indicated. *p*-values are from one-way ANOVA for continuous variables and ^†^ χ^2^ test (or Fisher’s exact test when appropriate) for categorical variables. Boldface indicates *p* < 0.05.

**Table 2:** Lipid profiles and CAC measures (Agatston score and CAC presence). (lipid subset, *n* = 636: A = 235, B = 134, AB = 70, O = 197).

Characteristic (n)	A (235)	B (134)	AB (70)	O (197)	*p*-Value
Total cholesterol, mg/dL	201 ± 35	199 ± 37	197 ± 36	198 ± 35	0.66
Triglyceride, mg/dL	139 ± 105	138 ± 110	135 ± 82	147 ± 99	0.57
HDL-cholesterol, mg/dL	57 ± 15	58 ± 15	56 ± 14	53 ± 13	0.029
LDL-cholesterol, mg/dL	118 ± 30	121 ± 33	116 ± 29	122 ± 31	0.31
LDL/HDL ratio	2.18 ± 0.79	2.08 ± 0.88	2.10 ± 0.68	2.46 ± 0.82	0.019
Blood glucose, mg/dL	109 ± 30	110 ± 33	108 ± 21	111 ± 33	0.65
Creatinine, mg/dL	0.77 ± 0.39	0.76 ± 0.42	0.75 ± 0.46	0.78 ± 0.19	0.21
Agatston score, median	7.2	0.0	0.0	19.8	0.14 ^†^
CAC present (Agatston >0), n (%)	122 (51.9)	61 (45.5)	31 (44.3)	106 (53.8)	0.33 ^‡^

**Abbreviations:** CAC, coronary artery calcium; HDL-C, high-density lipoprotein cholesterol; LDL-C, low-density lipoprotein cholesterol; TG, triglycerides; CCTA, coronary computed tomographic angiography; ABO, ABO blood group. **Notes:** Values are mean ± SD unless otherwise indicated. *p*-values were calculated using one-way ANOVA for continuous variables, except ^†^ Kruskal–Wallis test for Agatston score. ^‡^ CAC presence was compared using the χ^2^ test. Boldface indicates *p* < 0.05.

**Table 3:** O vs. Non-O Comparisons of Risk Factors, Lipids, and CAC (Overall Cohort and Lipid Subset). Overall cohort: O = 268, non-O = 597. Lipid subset: O = 197, non-O = 439.

Characteristic	O	Non-O	*p*-Value
Overall cohort (*n* = 865)			
Age, years	68 ± 12	67 ± 12	0.16
Height, cm	160 ± 9	161 ± 10	0.15
Weight, kg	62 ± 13	62 ± 13	0.87
BMI, kg/m^2^	24.3 ± 3.8	24.1 ± 3.2	0.82
SBP, mmHg	146 ± 22	145 ± 22	0.47
DBP, mmHg	88 ± 14	86 ± 13	0.03 *
Family history, %	27	25	0.76
Hypertension, %	63	57	0.04 *
Dyslipidemia, %	64	61	0.16
Diabetes, %	21	21	0.51
Current smoking, %	13	15	0.16
CKD, %	2	2	0.32
CAC present (Agatston > 0), %	53	51	0.38
Agatston score, median	14.3	5.1	0.20
Lipid subset (*n* = 636)			
Total cholesterol, mg/dL	198 ± 36	199 ± 36	0.73
Triglycerides, mg/dL	146 ± 98	139 ± 105	0.28
HDL-cholesterol, mg/dL	53 ± 13	57 ± 15	0.01 **
LDL-cholesterol, mg/dL	122 ± 32	118 ± 32	0.23
LDL/HDL ratio	2.46 ± 0.82	2.24 ± 0.83	0.02 *

**Abbreviations:** BMI, body mass index; SBP, systolic blood pressure; DBP, diastolic blood pressure; CKD, chronic kidney disease; CAC, coronary artery calcium; HDL-C, high-density lipoprotein cholesterol; LDL-C, low-density lipoprotein cholesterol; ABO, ABO blood group. **Notes:** Blood pressure and clinical characteristics are from the overall cohort (*n* = 865; O = 268; non-O = 597). Lipid variables are from the lipid subset (*n* = 636; O = 197; non-O = 439). Values are mean ± SD unless otherwise specified; Agatston score is median. * *p* < 0.05; ** *p* < 0.01.

**Table 4:** Multivariable predictors of any CAC (Agatston > 0); adjusted ORs (95% CI).

Predictor	aOR (95% CI)	*p*-Value
Age, per 10 years	1.65 (1.48–1.84)	<0.001 ***
Male sex	1.58 (1.26–1.98)	<0.001 ***
BMI, per kg/m^2^	1.02 (0.99–1.05)	0.18
Current smoking	1.21 (0.93–1.57)	0.15
Hypertension	1.34 (1.07–1.68)	0.01 *
Diabetes mellitus	1.29 (1.02–1.64)	0.03 *
LDL-cholesterol, per 10 mg/dL	1.05 (1.00–1.10)	0.06
HDL-cholesterol, per 10 mg/dL	0.95 (0.87–1.03)	0.22
Triglyceride, per 10 mg/dL	1.01 (1.00–1.02)	0.09
eGFR, per 10 mL/min/1.73 m^2^	0.96 (0.90–1.02)	0.17
ABO (O vs. non-O)	1.05 (0.86–1.28)	0.58

*n* = 636. * *p* < 0.05; *** *p* < 0.001. **Abbreviations:** aOR, adjusted odds ratio; CI, confidence interval; CAC, coronary artery calcium; CCTA, coronary computed tomographic angiography; eGFR, estimated glomerular filtration rate.

**Table 5:** Multivariable predictors of ≥50% stenosis on CCTA; adjusted ORs (95% CI).

Predictor	aOR (95% CI)	*p*-Value
Age, per 10 years	1.40 (1.25–1.58)	<0.001 ***
Male sex	1.52 (1.18–1.96)	0.001 **
BMI, per kg/m^2^	1.01 (0.98–1.04)	0.52
Current smoking	1.18 (0.89–1.56)	0.25
Hypertension	1.45 (1.14–1.85)	0.003 **
Diabetes mellitus	1.55 (1.20–2.00)	0.001 **
LDL-cholesterol, per 10 mg/dL	1.04 (0.99–1.09)	0.10
HDL-cholesterol, per 10 mg/dL	0.88 (0.79–0.99)	0.036 *
Triglyceride, per 10 mg/dL	1.00 (0.99–1.01)	0.48
eGFR, per 10 mL/min/1.73 m^2^	0.97 (0.91–1.04)	0.40
ABO (O vs. non-O)	0.98 (0.78–1.23)	0.87

*n* = 636. * *p* < 0.05; ** *p* < 0.01; *** *p* < 0.001. **Abbreviations:** aOR, adjusted odds ratio; CI, confidence interval; CAC, coronary artery calcium; CCTA, coronary computed tomographic angiography; eGFR, estimated glomerular filtration rate.

## Data Availability

The data presented in this study are available on request from the corresponding author. The data are not publicly available due to privacy and ethical restrictions (patient confidentiality and Institutional Review Board requirements).

## Abbreviations

The following abbreviations are used in this manuscript:

ABO	ABO blood group
AHA	American Heart Association
BMI	Body Mass Index
BP	Blood Pressure
SBP	Systolic Blood Pressure
DBP	Diastolic Blood Pressure
CAD	Coronary Artery Disease
CAC	Coronary Artery Calcium
CACS	Coronary Artery Calcium Score/Scoring
CCTA	Coronary Computed Tomographic Angiography
CKD	Chronic Kidney Disease
CI	Confidence Interval
eGFR	estimated Glomerular Filtration Rate
ECG	Electrocardiogram/Electrocardiography
FVIII	Coagulation Factor VIII
GWAS	Genome-Wide Association Study
HDL-C	High-Density Lipoprotein Cholesterol
HR	Heart Rate
HU	Hounsfield Unit
KDIGO	Kidney Disease: Improving Global Outcomes
LDL-C	Low-Density Lipoprotein Cholesterol
ORNGSP	Odds RatioNational Glycohemoglobin Standardization Program
aOROR	Adjusted Odds RatioOdds Ratio
SCCTaOR	Society of Cardiovascular Computed TomographyAdjusted Odds Ratio
SDSCCT	Standard DeviationSociety of Cardiovascular Computed Tomography
SPSSSD	Statistical Package for the Social SciencesStandard Deviation
vWFSPSS	von Willebrand factorStatistical Package for the Social Sciences
vWF	von Willebrand factor
